# Personal life and working conditions of trainees and young specialists in clinical microbiology and infectious diseases in Europe: a questionnaire survey

**DOI:** 10.1007/s10096-017-2937-4

**Published:** 2017-02-24

**Authors:** A. E. Maraolo, D. S. Y. Ong, J. Cortez, K. Dedić, D. Dušek, A. Martin-Quiros, P. J. Maver, C. Skevaki, E. Yusuf, M. Poljak, M. Sanguinetti, E. Tacconelli, The Trainee Association of the European Society of Clinical Microbiology and Infectious Diseases (ESCMID)

**Affiliations:** 1 Department of Clinical Medicine and Surgery, Section of Infectious Diseases, University of Naples “Federico II”, Naples, Italy; 2 Department of Medical Microbiology, University Medical Center Utrecht, Utrecht, The Netherlands; 3 Infectious Diseases Department, Centro Hospitalar e Universitário de Coimbra, Coimbra, Portugal; 4 CISA, Health Research Centre of Angola, Caxito, Angola; 5 Microbiology Department, Cantonal Hospital “Dr. Irfan Ljubijankic”, Bihac, Bosnia and Herzegovina; 6 University Hospital for Infectious Diseases “Dr. Fran Mihaljevic”, Zagreb, Croatia; 7 Emergency Department, Instituto de Investigación del Hospital Universitario La Paz, Madrid, Spain; 8 Institute of Microbiology and Immunology, Faculty of Medicine, University of Ljubljana, Ljubljana, Slovenia; 9 University Hospital Giessen and Marburg GmbH, Philipps University, Marburg, Germany; 10 Department of Medical Microbiology, Universitair Ziekenhuis Antwerpen, University of Antwerp, Edegem, Belgium; 11 Institute of Microbiology, Università Cattolica del Sacro Cuore, Rome, Italy; 12 Infectious Diseases, University Hospital Tübingen, DZIF Center, Tübingen, Germany

## Abstract

The purpose of this investigation was to assess the balance between the personal and professional lives of trainees and young European specialists in clinical microbiology (CM) and infectious diseases (ID), and determine differences according to gender, country of training, workplace and specialty. The Steering Committee of the Trainee Association of the European Society of Clinical Microbiology and Infectious Diseases (ESCMID) devised a questionnaire survey consisting, beyond the demographic questions, of nine yes/no questions, 11 Likert scale self-evaluations and one open-response item on parenthood, working conditions, quality of life, alcohol consumption and burnout. This anonymous survey in English was held between April and July 2015 among European CM/ID trainees and young specialists (<3 years after training completion). Responses from 416 participants with a mean age of 32 years [standard deviation (SD) 5 years] were analysed. Females and physicians from Northern/Western Europe (NWE) benefit more from paternity/maternity leaves even during training than their counterparts. Among all respondents, only half of breastfeeding mothers enjoyed the benefit of working hours flexibility. Only two-thirds of respondents found their working environment stimulating. In comparison to colleagues from other parts of Europe, trainees and young specialists from Southern/Eastern Europe (SEE) had less frequent regular meetings with mentors/supervisors and head of departments where trainees’ issues are discussed. Also, physicians from SEE were more frequently victims of workplace mobbing/bullying in comparison to those from other regions. Finally, multivariate analysis showed that female gender, SEE region and ID specialty were associated with burnout feelings. Female gender and country of work from SEE largely determine satisfactory working conditions, the possibility of parenthood leaves, amount of leisure time, mobbing experiences and burnout feelings among European CM/ID trainees and young specialists.

**Electronic supplementary material:**

The online version of this article (doi:10.1007/s10096-017-2937-4) contains supplementary material, which is available to authorized users.

## Introduction

Achieving balance between personal and professional life is extremely important for each individual. Physicians are among the most susceptible individuals failing to achieve this balance due to heavy work demands, long working hours, shift work (including night shifts) and staff shortages. Imbalance between professional and personal life is a well-known risk factor for work stress and burnout [1, 2].

The consequences of long periods of excessive work under stress conditions and burnout could have serious outcomes for the wellness of individual physicians [3]. Burnout is a very important predictor in career satisfaction as well. In addition, it can be one of the core elements underlying the strikingly high prevalence of depression or depressive symptoms (28.8%) among resident physicians [4].

As of 2013, more than 180 reports have documented that physicians are highly dissatisfied with their jobs [5]. In the Unites States, a recent survey showed that physicians (from all specialty disciplines) early in their career had the lowest career satisfaction, greatest rates of work–home conflicts, more difficulty resolving work–home conflicts in a manner that allowed both work and home responsibilities to be met, and greater depersonalisation in comparison to physicians more advanced in their careers [6]. To the best of our knowledge, no study has been published in peer-reviewed literature that addresses these important questions among trainees and young specialists in clinical microbiology (CM) and infectious diseases (ID) in Europe, whereas surveys with similar aims were conducted among European trainees in other specialties, such as surgical oncology [7].

Therefore, the Trainee Association of the European Society of Clinical Microbiology and Infectious Diseases (TAE), whose paramount goal is to improve collaboration among trainees and to improve their learning path during residency, devised a questionnaire survey to assess several aspects related both to training and to the balance between personal and professional life (work–life balance). The results addressing issues such as training satisfaction rate and the adequacy of the training itself are published elsewhere [8]. The present paper focuses on different aspects of work–life balance, including parenthood, working conditions, quality of life, activities during leisure time, alcohol consumption and burnout.

## Materials and methods

### Survey strategy

The present study is a survey conceived by the Steering Committee of the TAE on the quality of life (QoL) perceived during medical postgraduate training across Europe. A pilot phase involving 32 participants from different European countries was conducted in order to assess the feasibility and reproducibility of the questionnaire, leading to the necessary amendments. The final survey was available online from 26 April to 13 July 2015, through the open source software Lime Survey (Lime Survey GmbH, Hamburg, Germany) addressing several aspects of trainees’ QoL: work flexibility for physicians becoming parents during training, workplace mobbing experience (i.e. bullying of an individual by a group; it concerns repetitive behaviours by peers and superiors that makes the workplace and daily activities of the subject uncomfortable), burnout feelings, adequate trainee representation on boards where the policy on training conditions is discussed, presence of a stimulating work environment, activities during free time and alcohol consumption. The survey was aimed at European trainees and young specialists (<3 years after training completion) in CM and ID, so as to have a reliable and current overview of the actual conditions of their training. The survey was promoted during the ESCMID’s European Congress of Clinical Microbiology and Infectious Diseases (ECCMID) annual meeting attracting more than 10,000 participants, on the ESCMID website, via TAE national contact persons and through the ESCMID members’ mailing list. Before starting to fill out the questionnaire survey, every respondent was informed about the purpose of the survey and that anonymity would be ensured.

The general purpose was to determine possible differences across the above-mentioned QoL domains according to four variables: gender, country (not referring to nationality but to the place of training), workplace (university or non-university environment) and specialty. Of note, participation of young physicians from non-European countries was allowed, as ESCMID membership is not restricted to European countries. In detail, because of only a small number of entries from the majority of countries, the five geographic categories used regularly by the ESCMID [9] have been turned into two, Northern/Western Europe (NWE, including Northern and Western Europe) and Southern/Eastern Europe (SEE, including Southern, South-Eastern and South-Western Europe), then adding an all-encompassing category (EEU, namely Extra-Europe) for all the non-European countries (online supplementary file 1); regarding the fourth variable, participants have been labelled as trainees/young specialists in ID, in CM, in both ID/CM (in a few countries, CM and ID exist as a combined specialty) and in any other field (another all-encompassing category of physicians neither ID nor CM).

### Questionnaire domains

The survey included, beyond demographic questions, nine yes/no questions, 11 Likert scale self-evaluations and one open-response item on the following topics (all answers, including the ones from young specialists, are related to the training period):

- Socio-demographic information such as gender, country of work, workplace, specialty as previously explained and marital status;
- Parenthood issues during training, with a subjective assessment of the way the training rules fit with their parental needs (five-point Likert scale, 1 = completely dissatisfied, 5 = completely satisfied);
- The presence of trainees’ representative(s) on the board(s) wherein training policies are discussed and the regular implementation of meetings of trainees with their mentors and heads of department;
- Self-assessment of the quality of the training work environment, whether it is stimulating or not;
- Leisure time, namely favourite activities (e.g. fitness/sports, family, cinema/theatre, reading/taking courses, other) and the possibility to perform physical exercise as well as volunteer work;
- Problems in carrying out daily job-related tasks, consisting of three items (three-point Likert scale, 1 = does not present a problem, 3 = this presents a big problem) about unfriendly colleagues, too many management commitments, too many working hours, increasing computerisation, low salary and hostile patients;
- Mobbing experience (yes/no), namely exposure to threats to professional status and to personal standing (such as motiveless menaces to be downgraded or to be rejected at the final exams), isolation and destabilisation [10];
- Burnout (defined as a pathological syndrome in which emotional depletion and maladaptive detachment occur because of prolonged occupational stress), consisting of four items (five-point Likert scale, 1 = never, 5 = very often) upon how often respondents feel they are achieving less than deserved, frustrated, misunderstood/unappreciated, worn out [11];
- Alcohol consumption, meaning how frequently a glass of alcohol is consumed (Likert scale 0–4, 0 = never, 4 = 4 glasses or more a week);
- Subjective assessment of overall QoL, namely the necessity to work during free time to fulfil work demands (0–4 Likert scale, 0 = never, 4 = nearly every day), whether the workload fits with social and family commitments (0–3 Likert scale, 0 = not very well, 3 = well) and whether respondents regret the specialty they have chosen.

### Statistical analysis

The results were collected by proMENTE Social Research (Sarajevo, Bosnia and Herzegovina), which prepared the database for further evaluation and in-depth examination. The analysis was performed by the members of Steering Committee of the TAE. Categorical variables were summarised by frequencies and percentages, and continuous variables were summarised by means and standard deviation (SDs). The responses to Likert scale questions were deemed to be equidistant and symmetric, and summarised as mean and SD. The continuous variables were investigated by separate factorial multivariate (by using Wilk’s lambda test) and univariate analysis of variance; *F*- and *p*-values are reported along with partial eta square (η^2^) for estimation of effect size. Categorical variables were analysed through Chi-square tests, and with Fisher’s exact test when appropriate. Statistical significance was set at *p* < 0.05. When necessary, reliability analysis by a Cronbach’s alpha test was performed, in order to understand whether questions about certain domains all measured the same latent variable coherently: only values of or superior to 0.7 have been considered satisfactory [12]. All analyses were carried out by using IBM SPSS Statistics for Windows Version 23.0 (IBM Corp., Armonk, NY).

## Results

Data from 416 participants were analysed. Table 1 illustrates the main characteristics of the respondents participating in the survey. The mean age of respondents was 32 years (SD 5 years). Most respondents came from Italy (*n* = 50; 12%), Croatia (*n* = 37; 9%), Spain (*n* = 35; 8%), Portugal (*n* = 34; 8%), France (*n* = 33; 8%), the Netherlands (*n* = 32; 8%) and Turkey (*n* = 26; 6%).

**Table 1:** Main characteristics of the participants

Category		Absolute numbers and percentages (%)
Gender	Males	157 (37.7)
	Females	259 (62.3)
Country (not of origin but where respondents work)	NWE	146 (35.1)
	SEE	236 (56.7)
	EEU	34 (8.2)
Speciality	ID t/ys	155 (37.3)
	CM t/ys	191 (45.9)
	ID/CM t/ys	44 (10.6)
	Other	26 (6.3)
Workplace	University centre	319 (76.7)
	Non-university centre	97 (23.3)

*NWE* Northern/Western Europe, *SEE* Southern/Eastern Europe, *EEU* Extra-Europe, *t/ys* trainees/young specialists

### Parenthood

Respondents were divided into three categories: single, in a relationship, but not living together with the stable partner, and cohabitant (married or not married) (online supplementary file 2). Respondents working in NWE are more often married or cohabitant. No differences were observed concerning gender, speciality and workplace. In total, 128/416 (30.8%) participants stated they had a child during their training: 45/157 (28.7%) men and 83/259 (32.0%) women (*p* = 0.469). A further analysis in this subgroup showed that female physicians from NWE benefit more from paternity/maternity leaves during training than their counterparts (Table 2): overall, 87/128 (68.0%) respondents who became parents stated that they had paternity or maternity leave (51.1% and 77.1%, respectively). Moreover, the evaluation of working hours flexibility for breastfeeding mothers showed that 43/87 (49.4%) enjoyed this benefit, which was not different across countries (*p* = 0.068), workplace (*p* = 0.516) or speciality (*p* = 0.386). Finally, the assessment of self-perceived satisfaction regarding the general flexibility guaranteed to parents during training showed only differences for gender: men were more satisfied than women (Table 2). No interaction between the variables was found.

**Table 2:** Allowed leaves during training for physicians who became parents and satisfaction about the flexibility to take care of their newborns during their training

	Parenthood leave, no. (% yes)	Satisfaction, mean (SD)	Satisfaction univariate analysis*		
			*F*	*p*	Partial η^2^
Gender					
Male (*n* = 45)	23 (51.1)	3.47 (0.99)	(1,126)	**0.038**	0.034
Female (*n* = 83)	64 (77.1)	3.05 (1.13)	4.38		
*p*	**0.003**				
Country of work					
NWE (*n* = 58)	47 (81)	3.24 (1.08)	(1,125)	0.826	0.003
SEE (*n* = 60)	36 (60)	3.13 (1.13)	0.19		
EEU (*n* = 10)	4 (40)	3.30 (1.06)			
*p*	**0.007**				
Workplace					
University centre (*n* = 101)	69 (68.3)	3.13 (1.11)	(1,126)	0.184	0.014
Non-university centre (*n* = 27)	18 (66.7)	3.34 (1.01)	1.78		
*p*	0.870				
Speciality					
ID t/ys (*n* = 50)	37 (74)	3.00 (0.99)	(1,124)	0.219	0.035
CM t/ys (*n* = 60)	41 (68.3)	3.40 (1.11)	1.497		
ID/CM t/ys (*n* = 8)	3 (37.5)	3.25 (1.17)			
Other (*n* = 10)	6 (60)	2.90 (1.37)			
*p*	0.209				

*NWE* Northern/Western Europe, *SEE* Southern/Eastern Europe, *EEU* Extra-Europe, *t/ys* trainees/young specialists

*Satisfaction was expressed on a five-point Likert scale: completely dissatisfied (=1), dissatisfied (=2), neither satisfied nor dissatisfied (=3), satisfied (=4) and completely satisfied (=5)

### Representation and work environment

Only the country of work was found to be associated with the presence of trainees’ representatives during regular meetings with mentors and head of departments in which trainees’ issues are discussed: physicians from SEE showed the lowest values (Table 3). Overall, 128/416 (31%) found their work environment not stimulating. Physicians from SEE perceived their work environment during training the least stimulating.

**Table 3:** Representation, work environment, leisure time and mobbing experience

	Representation, yes (%)	Meetings with mentors/HD, yes (%)	Stimulating environment, yes (%)	Physical activity, yes (%)	Volunteer work, yes (%)	Mobbing experience, yes (%)
Gender						
Male (*n* = 157)	NA	NA	115 (73.7)	106 (40.2)	55 (35.0)	28 (17.8)
Female (*n* = 259)			173 (66.8)	158 (61.0)	80 (30.9)	63 (24.3)
*p*			0.138	0.181	0.382	0.121
Country of work						
NWE (*n* = 146)	85 (58.2)	85 (58.2)	131 (89.7)	114 (78.1)	38 (26.0)	19 (13.0)
SEE (*n* = 236)	74 (31.4)	82 (34.7)	130 (55.1)	129 (54.7)	77 (32.8)	68 (28.8)
EEU (*n* = 34)	17 (50.0)	25 (73.5)	27 (79.4)	21 (61.8)	20 (58.8)	4 (11.8)
*p*	**<0.001**	**<0.001**	**<0.001**	**<0.001**	**0.001**	**<0.001**
Workplace						
University centre (*n* = 319)	142 (44.5)	151 (47.3)	223 (69.9)	206 (64.6)	98 (30.8)	74 (23.2)
Non-university centre (*n* = 97)	34 (35.1)	41 (42.3)	65 (67.7)	58 (59.8)	37 (38.1)	17 (17.5)
*p*	0.102	0.416	0.682	0.392	0.220	0.237
Speciality						
ID t/ys (*n* = 155)	61 (39.4)	60 (38.7)	104 (67.1)	93 (60.0)	53 (34.2)	35 (22.6)
CM t/ys (*n* = 191)	88 (46.1)	97 (50.8)	138 (72.6)	129 (67.5)	58 (30.4)	35 (18.3)
ID/CM t/ys (*n* = 44)	17 (38.6)	19 (43.2)	30 (68.2)	28 (63.6)	12 (27.3)	16 (36.4)
Other (*n* = 26)	10 (38.5)	16 (61.5)	16 (61.5)	14 (53.8)	12 (46.2)	5 (19.2)
*p*	0.559	0.052	0.550	0.361	0.340	0.073

*HD* Head of department, *NA* not applicable, *NWE* Northern/Western Europe, *SEE* Southern/Eastern Europe, *EEU* Extra-Europe, *t/ys* trainees/young specialists

### Leisure time

Regular physical activity, defined as at least 1 h of gym/running every other day, was reported by 264 (44%) respondents. Trainees from SEE had the least physical activity (Table 3), whereas participants from the same region reported the highest participation in volunteer work. There were no other statistically significant differences regarding physical activity. Among the principal activities during free time, ‘time with family’ was preferred by the majority of respondents: 191/416 (45.9%). Interestingly, this was also the only activity that was different across gender: 61/157 (39.9%) males spent time with family versus 130/259 (50.2%) females (*p* = 0.009). Other favourite activities performed by respondents were: fitness/sport 85/416 (20.4%), going to the cinema/theatre 58/416 (13.9%) and taking recreational courses 38/416 (9.1%).

### Mobbing experience

Altogether, 91/416 (21.9%) respondents claim they have experienced mobbing during training. Physicians from SEE reported more mobbing in comparison to those from other regions (Table 3). Although not statistically significant, there was a tendency for more mobbing experience among females than in males and more in people working in university centres compared to physicians working at other sites.

### Burnout

Respondents were asked to fill in a four-item questionnaire on the topic of burnout (Table 4), of which the internal validity was confirmed by a high Cronbach’s alpha (0.824). The multivariate analysis (online supplementary file 3) showed a significant difference for gender, country of work and specialty. Females have more major burnout feelings than males, especially feelings of ‘achieving less than deserved’ and ‘feeling worn out.’ Physicians from SEE have more intense burnout feelings in all of the four domains. ID trainees/young specialists showed major burnout feelings with respect to the domains ‘achieving less than deserved’ and ‘frustration.’

**Table 4:** Burnout feelings

Category		No.	Achieving less than deserved	Frustration	Feeling unappreciated	Feeling worn out
Gender	Male	157	2.67 (0.99)	2.94 (1.07)	2.40 (1.00)	2.67 (0.99)
	Female	259	2.95 (0.96)	2.99 (1.00)	2.55 (0.98)	2.95 (0.96)
Country of work	NWE	146	2.88 (0.94)	2.74 (0.93)	2.08 (0.83)	2.55 (0.85)
	SWE	236	3.43 (1.11)	3.15 (1.06)	2.72 (1.01)	3.03 (1.00)
	EEU	34	2.85 (1.05)	2.71 (0.97)	2.70 (0.85)	2.79 (1.04)
Workplace	University centre	319	3.27 (1.07)	3.04 (1.01)	2.56 (1.01)	2.88 (0.97)
	Non-university centre	97	2.95 (1.08)	2.73 (1.04)	2.28 (0.87)	2.71 (0.99)
Speciality	ID t/ys	155	3.41 (1.12)	3.21 (1.07)	2.64 (1.07)	3.02 (1.02)
	CM t/ys	91	3.13 (1.05)	2.79 (0.93)	2.39 (0.91)	2.67 (0.91)
	ID/CM t/ys	44	2.82 (1.04)	2.77 (1.03)	2.32 (0.88)	2.77 (0.94)
	Other	26	3.00 (0.89)	3.23 (1.11)	2.65 (1.19)	3.19 (1.02)
Total		416	3.19 (1.08)	2.97 (1.02)	2.49 (0.99)	2.84 (0.98)

*NWE* Northern/Western Europe, *SEE* Southern/Eastern Europe, *EEU* Extra-Europe, *t/ys* trainees/young specialists

Data are presented as mean (standard deviation, SD)

All four items of burnout were expressed on a five-point Likert scale: never (=1), rarely (=2), sometimes (=3), often (=4) and very often (=5)

### Alcohol consumption

Statistically significant differences in the frequency of alcohol consumption were observed across gender and country of work: male physicians from NWE reported the highest alcohol consumption rates (Table 5).

**Table 5:** Frequency of alcohol consumption

Category		No.	Alcohol consumption, mean (SD)	Univariate analysis		
				*F*	*p*	Partial η^2^
Gender	Male	157	1.87 (1.16)	(1,414) 6.05	**0.014**	0.014
	Female	259	1.60 (1.00)			
Country of work	NWE	146	2.02 (1.03)	(1,413) 12.46	**<0.001**	0.057
	SWE	236	1.58 (1.01)			
	EEU	34	1.21 (1.27)			
Workplace	University centre	319	1.76 (1.06)	(1,414) 3.46	0.063	0.008
	Non-university centre	97	1.53 (1.06)			
Speciality	ID t/ys	155	1.81 (1.03)	(1,412) 1.497	0.283	0.009
	CM t/ys	91	1.61 (1.06)			
	ID/CM t/ys	44	1.64 (1.12)			
	Other	26	1.85 (1.23)			
Total		416	1.70 (1.07)			

*t/ys* Trainees/young specialists

Alcohol assumption was expressed on a five-point Likert scale: never (=0), once a month or less (=1), 2 to 4 times a month (=2), 2 to 3 times a week (=3) and 4 or more times a week (=4)

### Subjective assessment of overall quality of life

Physicians from university centres and ID trainees/young specialists reported the highest frequency of work during free time to fulfil work demands (Table 6). Regarding the issue of how workload fits with social and/or family commitments, differences were only found for country of work and speciality: physicians from EEU and CM residents/young specialists declared the highest impact of their work on social and family commitments.

**Table 6:** The need to work during free time to fulfill work demands and impact on social and family commitments

Category		No.	Work during free time, mean (SD)	Univariate analysis			Impact of work on personal commitment, mean (SD)	Univariate analysis		
				*F*	*p*	Partial η^2^		*F*	*p*	Partial η^2^
Gender	Male	157	2.64 (1.12)	(1,414) 0.05	0.831	0.000	2.29 (1.05)	(1,414) 1.04	0.309	0.003
	Female	259	2.62 (1.22)				2.19 (1.04)			
Country of work	NWE	146	2.59 (1.10)	(1,413) 0.70	0.496	0.003	2.39 (0.99)	(1,413) 6.59	**0.002**	0.031
	SWE	236	2.62 (1.25)				2.07 (1.05)			
	EEU	34	2.85 (1.05)				2.59 (1.08)			
Workplace	University centre	319	2.70 (1.15)	(1,414) 4.04	**0.032**	0.011	2.18 (1.03)	(1,414) 2.81	0.094	0.007
	Non-university centre	97	2.40 (1.25)				2.38 (1.09)			
Speciality	ID t/ys	155	2.97 (1.01)	(1,412) 1.497	**<0.001**	0.057	1.88 (0.95)	(1,412) 10.92	**<0.001**	0.074
	CM t/ys	91	2.38 (1.21)				2.50 (1.01)			
	ID/CM t/ys	44	2.41 (1.32)				2.34 (1.08)			
	Other	26	2.813 (1.23)				2.08 (1.20)			
Total		416	3.20 (1.09)				2.23 (1.05)			

*NWE* Northern/Western Europe, *SEE* Southern/Eastern Europe, *EEU* Extra-Europe, *t/ys* trainees/young specialists

The data represent the results of univariate analysis on how many respondents have to work during free time to fulfill work demands and impact of work on social and/or family commitments

The necessity for work during free time was expressed on a five-point Likert scale: never (=0), less often (=1), once or twice a month (=2), once or twice a week (=3) and nearly every day (=4). The impact of work on personal commitment was expressed on a four-point Likert scale: not very well (=0), not well (=1), well (=2) and very well (=3)

## Discussion

The major conclusions of our study are as follows: (i) gender differences exist across different dimensions in the personal lives among ID/CM European trainees; (ii) working conditions of an ID/CM trainee are dependent on the European region where the training has been performed; (iii) family time is the most appreciated activity during free time.

Our study showed that parenthood is perceived by a number of participants as having a significant negative impact on the professional career of young physicians, especially of our female colleagues. This is probably the result of an increased proportion of female medical graduates and, consequently, female trainees in both CM and ID in recent years in Europe [13, 14], and traditional opinion (still prevailing in the majority of European countries) that parenthood is mainly the mother’s responsibility. Additionally, about half of breastfeeding mothers in this study enjoyed the benefit of working hours flexibility, which can be a significant obstacle for an advanced and academic career, as previously shown by other studies [15, 16]. Thus, it might be wise to provide mentoring programmes, role models and flexible career structures, which can support female physicians. According to this survey, the satisfaction of having working hours flexibility as a parent during training was surprisingly higher among male physicians. It has been shown that parenthood also has an impact on an individual’s satisfaction with his or her career besides the impact on subjective career success [17].

Our data show that young physicians from SEE had less stimulation from their work environment, performed less physical activity during their free time, did voluntary work more often and had been subjected to more mobbing compared to their peers in NWE. Previous studies reported complaints of bureaucratic tasks, little time for education and poor support from tutors/supervisors in both Greece [18, 19] and Spain [20].

Even high-income countries could not offer the ideal work conditions to medical residents, as shown by the recent troubled negotiations in the United Kingdom between the government and junior doctors on a new contract entailing a quantitative improvement of staffing levels in hospitals, especially during nights and weekends [21]. The proposal stems from two premises that are attributed to a lack of personnel: the so-called ‘weekend effect,’ namely the presumed higher mortality risk for patients hospitalised on Saturdays and Sundays [22], and the delays to discharge from hospital (thereby increasing hospital length of stay and costs) [23]. The morale among UK young physicians is low, owing to overwhelming workloads, poor quality of training and lack of flexibility in working schedules [24]. Unhappy and unsatisfied doctors are associated with worse patient outcomes [24].

In our survey, this pattern of remarkable dissatisfaction especially concerns the participants from SEE (>50%). It is particularly worrisome that they report, besides a non-stimulating work environment, the lack of opportunities, e.g. including regular and official meetings with their tutors and heads of department to voice their malcontent, directly or through representatives. Advanced and multi-faceted mentorship programmes, including clinical, professional and academic components, with constant assistance and feedback, were much appreciated by residents [25]. Other worrisome aspects are the higher values trainees and young specialists from SEE report in terms of mobbing and burnout experience at work.

Burnout among healthcare professionals has become an important issue over the past several years, and it may be broadly defined as a state of mental and physical exhaustion due to work or caregiving activities [26]. The 22-item Maslach Burnout Inventory, tested also on medical residents [27], is the most commonly used questionnaire [28], although it is not the only tool to assess the main related domains such as exhaustion, depersonalisation and reduced personal accomplishment [26]. We devised a simplified Likert scale questionnaire that was statistically reliable after running a proper Cronbach’s alpha test. Burnout varies across different residency specialties, from 75% in obstetrics-gynaecology to 27% in family medicine [26]. Our survey notably states that females, physicians from SEE and ID physicians are often affected by this syndrome. Several factors are deemed important in contributing to residents’ burnout, such as time demands, difficult job situations, lack of control and work planning [26]. In a study conducted among Dutch residents, gender differences were also relevant [29]; in our study, women have more burnout feelings than men. Preventive and therapeutic approaches should include workplace-driven and individual-driven measures, such as workload modification, diversification of work duties and stress management training [26], but also take into account gender and the interference between home and work.

This survey emphasised family time as the most appreciated leisure activity. Indeed, many studies have shown that certain medical specialities, such as surgery, are highly incompatible with a satisfactory work–family balance [30–33]. The burden of family duties is disproportionally taken over by women, as identified in a recent survey among European CM/ID specialists [9].

An important limitation of this survey is the uncertainty of whether the participants adequately represent the entire population of CM/ID trainees in Europe. Unfortunately, reliable information on the number of CM/ID trainees in the majority of European countries is lacking, which hindered the estimation of the representativeness of our survey. Furthermore, this study is limited by the inherent limitations of opinion-based surveys. We did not combine these quantitative data with qualitative data in order to explore perceptions in parenthood, mobbing experience and burnout more in depth.

In conclusion, the first survey among European trainees in CM and ID shows heterogeneity in satisfaction on having an adequate work–life balance, which is known to be an important factor for the prevention of work stress and burnout, but also the quality of medical care.

## Electronic supplementary material

Below are the links to the electronic supplementary material.

ESM 1 (PDF 44 kb)

ESM 2 (PDF 37 kb)

ESM 3 (PDF 39 kb)
