# Supplementary material for: Personal life and working conditions of trainees and young specialists in clinical microbiology and infectious diseases in Europe: a questionnaire survey
Source: Eur J Clin Microbiol Infect Dis. 2017 Feb 24;36(7):1287–95. doi: 10.1007/s10096-017-2937-4 (PMC5495844; doi:10.1007/s10096-017-2937-4)
Supplement: Supplementary file 1 — (PDF 44 kb) [file 10096_2017_2937_MOESM1_ESM.pdf]

Online Supplementary File 1

ESCMID Definition of European Regions

**Western Europe**<br>• Austria (AT),<br>• Belgium (BE)<br>• France (FR)<br>• Germany (DE)<br>• Republic of Ireland (IE)<br>• Liechtenstein (LI)<br>• Luxembourg (LU)<br>• The Netherlands(NL)<br>• Switzerland (CH)<br>• United Kingdom (UK)	**South-Western Europe**<br>• Andorra (AD)<br>• Italy (IT)<br>• Malta (MT)<br>• Monaco (MC)<br>• Portugal (PT)<br>• San Marino (SM)<br>• Spain (ES)
**Northern Europe**<br>• Denmark (DK)<br>• Finland (FI)<br>• Iceland (IS)<br>• Norway (NO)<br>• Sweden (SE<br><br>**Eastern Europe**<br>• Armenia (AM)<br>• Azerbaijan (AZ)<br>• Belarus(BY)<br>• Czech Republic (CZ)<br>• Estonia (EE)<br>• Georgia (GE)<br>• Hungary (HU)<br>• Latvia (LV)<br>• Lithuania (LT)<br>• Moldova (MD)<br>• Poland (PL)<br>• Romania (RO)<br>• Russia (RU)<br>• Slovakia (SK)<br>• Ukraine (UA)	**South-Eastern Europe**<br>• Albania (AL)<br>• Bosnia and Herzegovina (BA)<br>• Bulgaria (BG)<br>• Croatia (HR)<br>• Cyprus(CY)<br>• Greece (GR)<br>• Israel (IL)<br>• Kosovo (KVM)<br>• Republic of Macedonia (MK)<br>• Montenegro (ME)<br>• Serbia (RS)<br>• Slovenia (SI)<br>• Turkey (TR)
