# Supplementary material for: Personal life and working conditions of trainees and young specialists in clinical microbiology and infectious diseases in Europe: a questionnaire survey
Source: Eur J Clin Microbiol Infect Dis. 2017 Feb 24;36(7):1287–95. doi: 10.1007/s10096-017-2937-4 (PMC5495844; doi:10.1007/s10096-017-2937-4)
Supplement: Supplementary file 2 — (PDF 37 kb) [file 10096_2017_2937_MOESM2_ESM.pdf]

Online Supplementary File 2.

Distribution of living status according to gender and country of work.

**Living Status**	**Gender (%)**		**Country of work (%)**		
	Males	Females	NWE	SEE	EEU
Single	41 (26.1)	68 (26.3)	30 (20.5)	64 (27.1)	15 (44.1)
Involved with someone but not cohabitant nor married	28 (17.8)	29 (11.2)	16 (11.0)	40 (16.9)	1 (2.9)
Married/cohabitant	88 (56.1)	162 (62.5)	100 (68.5)	132 (56.0)	18 (53.0)
Total	157	259	146	216	34
p	0.147		**0.007**		

NWE: Northern/Western Europe; SEE: Southern/Eastern Europe; EEU: Extra-Europe
