# Supplementary material for: Personal life and working conditions of trainees and young specialists in clinical microbiology and infectious diseases in Europe: a questionnaire survey
Source: Eur J Clin Microbiol Infect Dis. 2017 Feb 24;36(7):1287–95. doi: 10.1007/s10096-017-2937-4 (PMC5495844; doi:10.1007/s10096-017-2937-4)
Supplement: Supplementary file 3 — (PDF 39 kb) [file 10096_2017_2937_MOESM3_ESM.pdf]

Online Supplementary File 3

Univariate and multivariate analysis on burnout feelings

	Gender			Country of work			Workplace			Speciality		
**UNIVARIATE ANALYSIS**	F (1,414)	p	partial η^2	F (1,413)	p	partial η^2	F (1,414)	p	partial η^2	F (1,412)	p	partial η^2
**Achieving less than deserved**	8.12	**0.004**	0.019	14.31	**< 0.001**	0.065	8.12	**0.011**	0.019	4.54	**0.004**	0.032
**Frustration**	0.25	0.615	0.001	8.72	**< 0.001**	0.041	8.12	**0.009**	0.019	6.16	**< 0.001**	0.043
**Feeling unappreciated**	2.41	0.121	0.006	22.46	**< 0.001**	0.098	8.12	**0.016**	0.019	2.48	0.061	0.018
**Feeling worn-out**	8.21	**0.004**	0.019	10.70	**< 0.001**	0.054	8.12	**0.128**	0.019	4.99	0.002	0.035
**MULTIVARIATE ANALYSIS**	F (4,370)	p	partial η^2	F (8,740)	p	partial η^2	F (4,370)	p	partial η^2	F (12,979)	p	partial η^2
	4.60	**0.001**	0.047	2.07	**0.037**	0.022	1.14	0.339	0.012	2.01	0**.021**	0.021
